# Diagnostic accuracy of magnetic resonance imaging for nerve injury in obstetric brachial plexus injury: protocol for systematic review and meta-analysis

**DOI:** 10.1186/s13643-022-02037-9

**Published:** 2022-08-20

**Authors:** Claire Hardie, James Brooks, Ryckie Wade, Irvin Teh, Grainne Bourke

**Affiliations:** 1 Leeds Institute for Medical Research, University of Leeds, Leeds, UK; 2 Department of Plastic and Reconstructive Surgery, Leeds Teaching Hospitals Trust, Leeds, UK; 3 Leeds Institute of Cardiovascular and Metabolic Medicine, University of Leeds, Leeds, UK; 4 Department of Integrative Medical Biology, University of Umea, Umeå, Sweden

**Keywords:** Brachial plexus neuropathies, Magnetic resonance imaging, Birth injuries

## Abstract

**Background:**

Early and accurate clinical diagnosis of the extent of obstetric brachial plexus injury (OBPI) is challenging. The current gold standard for delineating the nerve injury is surgical exploration, and synchronous reconstruction is performed if indicated. Magnetic resonance imaging (MRI) is a non-invasive method of assessing the anatomy and severity of nerve injury in OBPI but the diagnostic accuracy is unclear.

The primary objective of this review is to determine the diagnostic accuracy of MRI in comparison to surgical brachial plexus exploration for detecting root avulsion in children under 5 with OBPI. The secondary objectives are to determine its’ diagnostic accuracy for detecting nerve abnormality and detecting pseudomeningocele(s) in this group.

**Methods:**

This review will be conducted according to the Preferred Reporting Items for Systematic Reviews and Meta-Analysis (PRISMA).We will include studies reporting the accuracy of MRI (index test) compared to surgical exploration (reference standard) in detecting any of the three target conditions (root avulsion, any nerve abnormality and pseudomeningocele) in children under five with OBPI. Case reports and studies where the number of true positives, false positives, true negatives and false negatives cannot be derived will be excluded. We plan to search PubMed, Embase and CENTRAL for relevant studies from database inception to 15 June 2022. We will also search grey literature (medRxiv, bioRxiv and Google Scholar) and perform forward and backward citation chasing. Screening and full-text assessment of eligibility will be conducted by two independent reviewers, who will then both extract the relevant data. The QUADAS-2 tool will be used to assess methodological quality and risk of bias of included studies by two reviewers independently. The following test characteristics for the target conditions will be extracted: true positives, false positives, true negatives and false negatives. Estimates of sensitivity and specificity with 95% confidence intervals will be shown in forest plots for each study. If appropriate, summary sensitivities and specificities for target conditions will be obtained via meta-analyses using a bivariate model.

**Discussion:**

This study will aim to clarify the diagnostic accuracy of MRI for detecting nerve injury in OBPI and define its clinical role.

**Systematic review registration:**

PROSPERO CRD42021267629.

**Supplementary Information:**

The online version contains supplementary material available at 10.1186/s13643-022-02037-9.

## Introduction

Obstetric brachial plexus injury (OBPI) is defined as flaccid paralysis of the upper limb at birth [1] and is typically caused by excessive traction applied to the neck during delivery. OBPI affects an estimated 0.4 to 2 children per 1000 births [2]. All nerve roots from C5-T1 can be damaged; however, C5 and C6 involvement (Erb’s palsy) is most frequent [3, 4]. Spontaneous recovery is common within the first 3 months of life; however, this recovery is incomplete in 10 to 30% of cases which leads to permanent loss of function [5, 6]. Whilst scoring systems such as the Active Movement Scale [7] can be used to aid in assessment, it is still difficult to clinically determine the extent of the injury so the diagnostic gold standard is surgical exploration of the brachial plexus. Surgical exploration is typically indicated if there is no or limited improvement in biceps or deltoid function at 3 months of age [8] or if the child has a low prognostic clinical score e.g. the Toronto score [7].

At the point of surgical exploration nerve reconstruction with transfer or grafts can be performed if required. Nerve transfer is used in cases of pre-ganglionic injury (root avulsion) which involves redirecting functioning nerves in the neck to supply the arm [9], and this type of injury conveys the worst prognosis. Post-ganglionic injuries are managed with nerve grafts which involve harvesting less important nerves (e.g. the sural nerve) and using them to restore continuity in the ruptured nerve. In both cases, early reconstructive nerve surgery is associated with better functional recovery [10].

Imaging can be used to evaluate OBPIs and support surgical decision making. Clinical practice has moved away from computed tomography myelography in the evaluation of OBPI and towards magnetic resonance imaging (MRI) [11] which has clear advantages when it comes to imaging children, such as the absence of ionising radiation and intrathecal contrast agent. Enhanced soft tissue visualisation, multiplanar reconstructions and the opportunity for quantative imaging also makes MRI a suitable modality. MRI is currently thought to be the best non-invasive test for imaging the brachial plexus and detecting nerve root avulsion [12], but has been demonstrated to have variable sensitivity (63–88%) and specificity (89–100%) for detecting pre-ganglionic injury and sensitivity 60–75% and specificity 89–100% for post-ganglionic injury [11, 13–16] when compared to surgical exploration. Pseudomeningoceles from leakage of cerebrospinal fluid can additionally be used as a surrogate marker of nerve root avulsion on MRI scans but again the reported accuracy of this is variable [17].

Due to the moderate sensitivity of MRI to detect nerve injury in OBPI, it cannot be relied on for prognostic information or to fully inform clinical decisions regarding need for exploratory surgery and type of surgery required. It is critical to differentiate between pre-ganglionic and post-ganglionic injuries due to the differing prognosis and surgical approaches for these injuries. There is a pressing need to define the characteristics of the nerve injury that has potential to recover with reconstructive surgery.

Surgical exploration of the brachial plexus is the reference standard for detecting root avulsion and other nerve abnormalities. The operation is performed under general anaesthesia and involves an incision in the supraclavicular fossa which extends towards the deltopectoral groove [17]. The operation allows direct inspection of the spinal nerve roots C4-T1. Additional intraoperative tests such as somatosensory-evoked potentials (SEPs) and bipolar motor nerve stimulation are included as part of the test. SEPs involve measuring cortical activity induced by applying pulses of varying frequency to the nerve roots. Avulsed nerves will not transmit signals to the brain meaning no activity is detected on an encephalogram. Bipolar nerve stimulation involves applying a current across the nerve which would normally cause the corresponding muscle to contract; however, in the case of avulsion, no muscle contraction is observed. These intraoperative tests aid surgeons in the diagnosis of root avulsion.

In OBPI, MRI is typically performed under general anaesthetic in infants with persistent upper limb functional limitations to evaluate the injury. The MRI acquisition varies in terms of the physical scanner used (manufacturer and model), field strength, pulse sequences, coil arrangement, gradients, postprocessing techniques and other factors—all of which will impact upon image quality and hence diagnostic accuracy. Variations can also arise due to the subjective nature of image interpretation. A radiologist reviews the images and either confirms or excludes the presence of avulsion, other nerve abnormality or pseudomeningocele. Positive findings for avulsion are detected by a lack of continuity or absence of the nerve root between the spinal cord and exit foramen [17]. Other nerve abnormalities than may be detected include nerve scarring, neuroma or rupture, and these can also be referred to as post-ganglionic nerve injuries [13, 15]. An abnormal contour of the dura and collection of dorsal extraspinous fluid is indicative of pseuomeningocele [18] and is considered a surrogate marker of root avulsion given that rupture of the dura mater suggests that the nerve root is also ruptured, although this has been disputed in some literature [19, 20]. The presence of one suspected avulsion is of equal importance to that of any number of avulsions, given that any avulsion would warrant surgical intervention. Due to the lower energy stretching forces that typically cause OBPI, other types of severe nerve injury apart from avulsion may also be present and can also require surgery e.g. nerve grafting. Avulsions, other nerve injury and pseudomeningoceles can occur at any spinal level from C4 to T2 and may, in rare cases, occur bilaterally.

### Why is it important to do this review?

OBPI is associated with significant life-long morbidity. Early clinical prognosis is challenging, but that may be improved with early surgical intervention. A highly sensitive and specific imaging modality that could clearly define the extent of the brachial plexus injury would enable earlier surgical intervention in those that require it. It would also facilitate complex operative planning. It also may reduce numbers of surgical explorations required in infants. MRI is beginning to be used in some centres to determine the extent of the nerve injury in OBPI, but the reliability in detecting root avulsion and other nerve abnormalities is uncertain [11, 21]. Assessing the current accuracy of MRI for obstetric brachial plexus injuries will help define its role clinically and suggest areas for development. This review aims to clarify the overall diagnostic accuracy by comparing MRI to surgical exploration as a reference standard.

### Objectives

The objectives have been formulated using the Population, Index test, Reference test, Diagnosis (PIRD) framework [22], see Table 1.

**Table 1:** Systematic review objectives defined using PIRD framework

Population	Children under 5 diagnosed with obstetric brachial plexus injury
**Index test**	MRI
**Reference test**	Surgical brachial plexus exploration
**Diagnoses of interest**	1. Root avulsion 2. Any type of nerve injury 3. Pseudomeningocele

#### Primary objective

1. To determine the diagnostic accuracy of MRI in comparison to surgical brachial plexus exploration for detecting root avulsion in children under 5 years old with obstetric brachial plexus injuries.

#### Secondary objectives

1. To determine the diagnostic accuracy of MRI in comparison to surgical brachial plexus exploration for for detecting nerve abnormality in children under 5 years old with obstetric brachial plexus injuries.
2. To determine the diagnostic accuracy of MRI in comparison to surgical brachial plexus exploration for detecting pseudomeningocele(s) as a surrogate marker of root avulsion in children under 5 years old with obstetric brachial plexus injuries.

## Methods

The Cochrane Handbook for Reviews of Diagnostic Test accuracy was used as guidance to write the methods, and the preferred reporting items for systematic reviews and meta-analyses (PRISMA-P) [23] guidelines were followed.

### Eligibility criteria

#### Types of studies

We will include all comparative test accuracy studies involving infants with obstetric brachial plexus injuries that report the findings of preoperative MRI in comparison to surgical exploration of the brachial plexus roots (single-gate design). Case reports will be excluded. Studies where the number of true positives, false positives, true negatives and false negatives cannot be derived will be excluded.

#### Participants

This review will include studies involving children under 5 years old with obstetric brachial plexus injuries. All injuries to the brachial plexus that occur during delivery will be included, irrespective of the aetiology (e.g. shoulder dystocia, forcep delivery). Bilateral injuries will also be included.

#### Index test

The index test will be MRI. The role of MRI will be to detect root avulsions, nerve abnormalities and pseudomeningoceles in children under 5 with OBPI.

#### Target condition

Avulsion of the roots of the brachial plexus is the target condition. The ability of MRI to differentiate between any number of root avulsions and no avulsions will be examined. The secondary target conditions are nerve abnormalities and pseudomeningocele.

#### Reference standard

Surgical exploration of the brachial plexus is the reference standard for detecting root avulsion, nerve abnormalities or pseudomeningocele.

### Information sources

#### Electronic searches

We plan to search Embase, PubMed and CENTRAL electronic databases from inception to 15 June 2022 with no restrictions. The medRxiv and bioRxiv preprint archives will also be searched using medrxivr [24]. GScraper will be used to further increase coverage by pulling hits from Google Scholar.

#### References from published studies

Citations will be imported and de-duplicated using EndNote. Forward and backward citation chasing will then be performed using CitationChaser [25].

#### Search strategy

The planned search strategy was formulated with a search strategist and is presented in Additional file 1.

#### Selection process

**Fig. 1:**
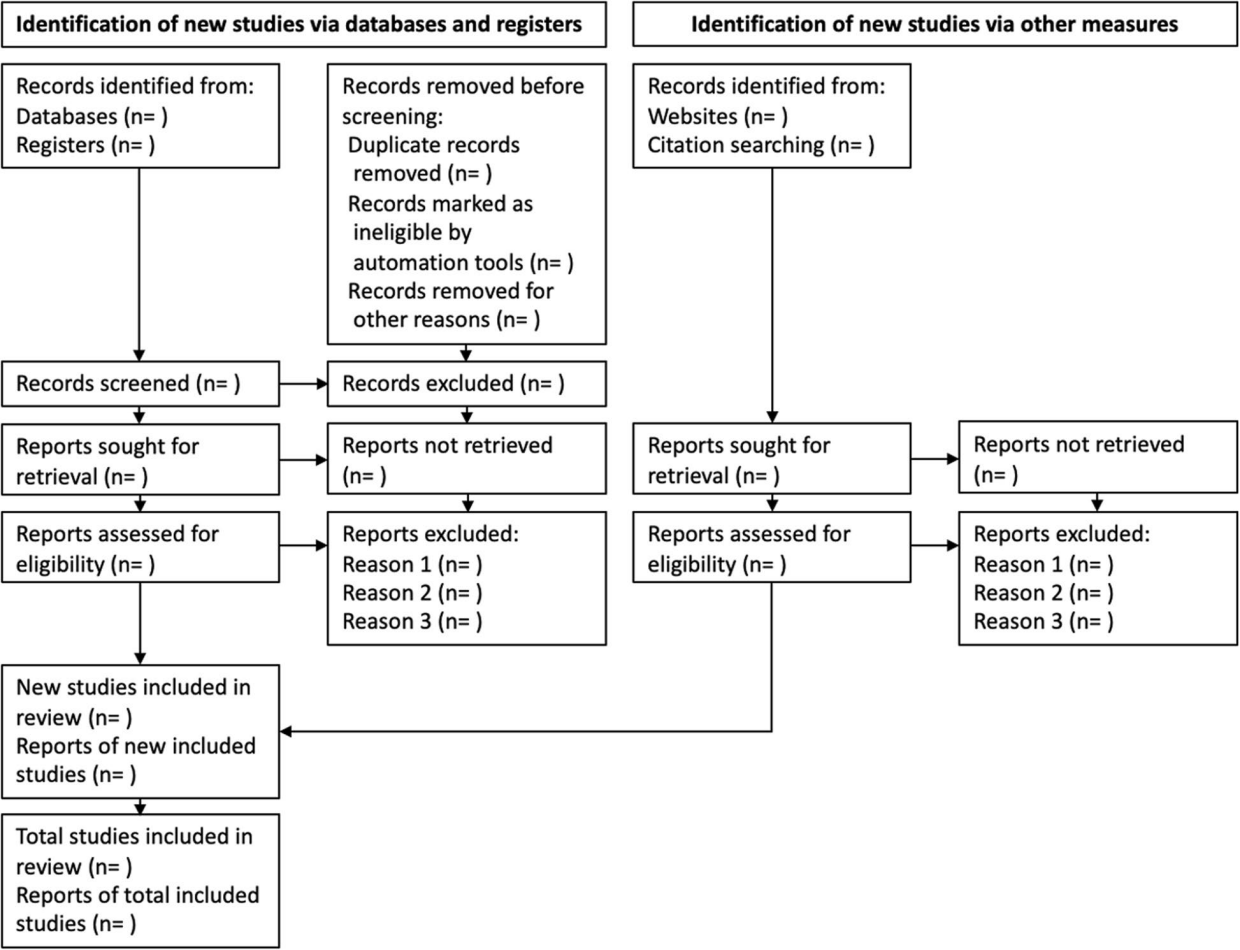
Prisma flow chart

Two reviewers (JB and CH) will independently apply the eligibility criteria to screen titles and abstracts for relevance. Two reviewers (JB and CH) will independently screen identified citations using Rayyan [26]. Full text of eligible studies will then be screened and subsequently labelled as included or excluded by two reviewers (CH and JB). Reasons for exclusion will be noted. Disagreements will be resolved by discussion with a third author (GB). A number of included studies at each stage will be shown in a PRISMA flow chart (Fig. 1).

#### Data collection process

For included studies, the following data will be extracted using an Excel spreadsheet developed by the reviewers and piloted. This will be done independently in duplicate by JB and CH. If data presented in the paper is unclear or further data is required, the corresponding author will be emailed to ask for this information.

#### Data items

The following data will be collected: study identifier; number of participants; sex; participants age at diagnosis, MRI scan and surgery; country of origin; time from birth to imaging and surgery; presence of clavicular/humeral fracture; type of MRI scanner used (including brand, model and field strength); pulse sequence. 2×2 contigency tables will be used to collect number of true positives, false positives, true negatives and false negatives relating to the detection of root avulsion, abnormal nerve or pseudomeningocele using MRI as the index test and surgical exploration as the reference test. The priority outcome is the detection of root avulsion at the brachial plexus as this is the is most important clinically. Detection of abnormal nerves and pseudomeningocele are secondary outcomes.

#### Study risk of bias assessment

The Quality Assessment of Diagnostic Accuracy Studies (QUADAS-2) [27] tool will be used to assess the risk of bias and strength of evidence of the eligible studies respectively at the study level. The assessment will be completed according to pre-defined review specific guiding comments (Additional file 2). Two authors (JB and CH) will independently carry out the assessment. Disagreements will be resolved by discussion with a third author (GB).

#### Effect measures (diagnostic accuracy measures)

Using the contingency tables, we will generate estimates of sensitivity and specificity for detection of each target condition for each study.

#### Synthesis methods

We will qualitatively describe the application of MRI for detection of nerve injury in OBPI. Analysis will be performed separately for each target condition (root avulsion, abnormal nerve and pseudomeningocele) with the nerve as the unit of analysis. Forest plots and receiver operating characteristic plots will be used to display estimates of sensitivity and specificity for each of the included studies as part of the preliminary analysis. These plots will be generated using MetaDTA [28]. As as secondary analysis the nerve unit data will be transformed to use the patient as the unit of analysis. Summary sensitivities and specificities will be obtained using a bivariate model for meta-analyses, providing data is sufficient [29].

#### Investigations of heterogeneity

Using Stata version 15, heterogeneity in the diagnostic accuracy of MRI will be examined using meta-regression or subgroup analyses if data permits. Variations in field strength is likely to be a source of heterogeneity [17] and will be investigated. Infants with OPBI who have not undergone surgical exploration (e.g. they are too unwell) may result in underestimation of the number false negatives which in turn could upwardly bias the sensitivity of MRI. Furthermore, the diagnostic accuracy of MRI could be biased by retrospective studies [30] which have recruited an unrepresentable sample of patients.

#### Sensitivity analysis

The impact of bias will be evaluated via sensitivity analyses. Studies with a high or unclear risk of bias as identified by the QUADAS-2 tool will be excluded.

#### Reporting bias assessment

Reporting bias will not be assessed given the lack of sensitive statistical methods.

#### Certainty assessment

Certainty assessment will be performed using the Grading of Recommendations Assessment, Development and Evaluation (GRADE) guidance for comparative test accuracy by two independent reviewers (CH and JB) [31].

## Discussion

OBPI can lead to significant morbidity both in terms of the physical implications (e.g. loss of function) and psychological impact [32, 33]. MRI offers a non-invasive method of visualising the brachial plexus which could potentially reduce the number of infants undergoing surgery and facilitate earlier treatment in those that require it. However, the reported sensitivity and specificity of MRI in detecting root avulsion and other nerve injuries is variable. A systematic review is required to determine the diagnostic accuracy of MRI in detecting nerve injury in infants with OBPI and define its role clinically. We anticipate variability in scanning techniques, scan reporting and surgical techniques and reporting of findings. There is likely to also be a mix of prospective and retrospective studies. We also anticipate possible presence of bias in included studies, for example non-consecutive recruiting or reporting only of positive findings MRI findings. The quality and variability of the included studies will be carefully evaluated using the methods described and results interpreted appropriately.

### Other information

#### Registration and protocol

PROSPERO registration CRD42021267629.

## Supplementary Information

**Additional file 1.** Search strategy.

**Additional file 2.** QUADAS-2.

## Data Availability

Data sharing is not applicable to this article as no datasets were generated or analysised. Materials (Search strategy and QUADAS-2 tool) are available in Additional file 1 and Additional file 2.

## Abbreviations

GRADE: Grading of Recommendations Assessment, Development and Evaluation
MRI: Magnetic resonance imaging
OBPI: Obstetric brachial plexus injury
QUADAS-2: Quality Assessment of Diagnostic Accuracy Studies
