# Supplementary material for: Diagnostic accuracy of magnetic resonance imaging for nerve injury in obstetric brachial plexus injury: protocol for systematic review and meta-analysis
Source: Syst Rev. 2022 Aug 20;11:173. doi: 10.1186/s13643-022-02037-9 (PMC9392905; doi:10.1186/s13643-022-02037-9)
Supplement: Supplementary file 1 — Additional file 1. Search strategy. [file 13643_2022_2037_MOESM1_ESM.docx]

## Additional file 1: Search strategy

Embase

1. Exp Brachial Plexus Neuropathy/
2. Brachial Plexus/
3. (brachial adj1 plex*).tw.
4. (brachial and plex*).ti.
5. infant/
6. newborn/
7. Newborn disease/
8. Child/
9. Obstetric delivery/
10. Infant/
11. Infant*.ti.
12. Infant*.ab. /freq=2
13. Child*.ti.
14. Child*.ab. /freq=2
15. Obstetrics/
16. Obstetric*.ti.
17. Obstetric*.ab. /freq=2
18. Neonat*.ti.
19. Neonat*.ab. /freq=2
20. Perinatal*.ti.
21. Perinatal.ab. /freq=2
22. Obstetrical Brachial Plexus Palsy/
23. Neonatal brachial plexus palsy/
24. ((neonat* or perinatal*) adj2 brachial plexus pals*).tw.
25. Birth injury/
26. Birth injury.ti.
27. Birth injury.ab. /freq=2
28. Paralysis, Obstetric/
29. (obstetric pals* or birth pals*).tw,kw.
30. ((obstetric or birth or perinatal*) adj3 brachial plexus injur*).tw,kw.
31. (klumpke* pals* or klumpke* paralysis).tw,kw.
32. (erb* adj2 paralysis).tw,kw.
33. (erb* adj2 pals*).tw,kw.
34. Exp Nuclear Magnetic Resonance Imaging/
35. ((magnetic resonance adj (imag* or scan* or tomograph*) or MRI or MR imag* or MR tomograph* or MRT or NMR or NMRI or fMRI or chemical shift imag*)).tw.
36. (MR?).ti,ab.
37. (magnetic AND resonance).ti,ab.
38. (NMR).ti,ab.
39. (neurogra*).ti,ab.
40. (DTI).ti,ab.
41. ((diffusion and tensor) and imaging).ti,ab.
42. 1 or 2 or 3 or 4
43. 5 or 6 or 7 or 8 or 9 or 10 or 11 or 12 or 13 or 14 or 15 or 16 or 17 or 18 or 19 or 20 or 21
44. 42 and 43
45. 22 or 23 or 24 or 25 or 26 or 27 or 28 or 29 or 30 or 31 or 32 or 33
46. 34 or 35 or 36 or 37 or 38 or 39 or 40 or 41
47. 44 or 45
48. 46 and 47

PubMed

1. Exp Brachial Plexus Neuropathy/
2. Brachial Plexus/
3. brachial adj1 plex*
4. brachial and plex*
5. infant*/
6. newborn/
7. Newborn disease/
8. Child/
9. Obstetric delivery/
10. Obstetric*
11. Neonat*
12. Perinatal*
13. Obstetrical Brachial Plexus Palsy/
14. Neonatal brachial plexus palsy/
15. (neonat* or perinatal*) adj2 brachial plexus pals*
16. Birth injury/
17. obstetric pals* or birth pals*
18. (obstetric or birth or perinatal*) adj3 brachial plexus injur*
19. klumpke* pals* or klumpke* paralysis
20. erb’s adj2 paralysis
21. erb’s adj2 pals*
22. Exp Nuclear Magnetic Resonance Imaging/
23. (magnetic resonance adj (imag* or scan* or tomograph*) or MRI or MR imag* or MR tomograph* or MRT or NMR or NMRI or fMRI or chemical shift imag*)
24. MR?
25. magnetic AND resonance
26. NMR
27. neurogra*
28. DTI
29. (diffusion and tensor) and imaging
30. 1 or 2 or 3 or 4
31. 5 or 6 or 7 or 8 or 9 or 10 or 11 or 12
32. 30 and 31
33. 13 or 23 or 24 or 25 or 26 or 27 or 28 or 29 or 30 or 31 or 32 or 21
34. 22 or 35 or 36 or 37 or 38 or 39 or 40 or 29
35. 33 or 34
36. 34 and 35
