# Supplementary material for: Diagnostic accuracy of magnetic resonance imaging for nerve injury in obstetric brachial plexus injury: protocol for systematic review and meta-analysis
Source: Syst Rev. 2022 Aug 20;11:173. doi: 10.1186/s13643-022-02037-9 (PMC9392905; doi:10.1186/s13643-022-02037-9)
Supplement: Supplementary file 2 — Additional file 2. QUADAS-2. [file 13643_2022_2037_MOESM2_ESM.docx]

## Additonal file 2: QUADAS-2

**QUADAS-2: Diagnostic Accuracy of Magnetic Resonance Imaging for Detecting Nerve Injury in Obstetric Brachial Plexus Injury**

**Study ID:**

Patients (setting, intended use of index test, presentation, prior testing):

Index test(s):

Reference standard and target condition:

**Domain 1: Patient Selection**

A. Risk of Bias

Describe the methods of patient selection:


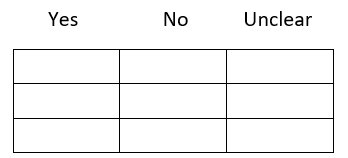


Was a consecutive or random sample of patients enrolled?
Was a case-control design avoided?
Did the study avoid inappropriate exclusions?

Could the selection of patients have introduced bias? **Low / High / Unclear***(Low = answered yes to all questions)
(High = answered no to at least one question)
(Otherwise, code as unclear)*

B. Concerns regarding applicability

Describe included patients (prior testing, presentation, intended use of index test and setting):

Is there concern that the included patients do not match the review question? **Low / High / Unclear***(Low = following factors were described and appropriate: prior tests, age at diagnosis, mode of delivery, clinical scoring, surgical method of exploration, field strength and pulse sequence(s) of MRI)
(Unclear = above factors were not described)
(High = multiple surgical explorations or MRIs were performed)*

**Domain 2: Index test**

A. Risk of bias

Describe the index test and how it was conducted and interpreted:


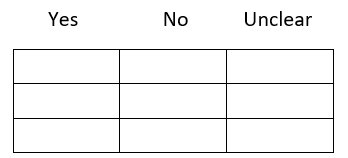
Were the MRI results interpreted without knowledge of the exploration?
If a threshold for either test was used, was it pre-specified?

Could the conduct or interpretation of the index test have introduced bias? **Low / High / Unclear**(Low = answered yes to both)
(High = answered no to both)
(Otherwise, code as unclear)

B. Concerns regarding applicability

Is there concern that the index test, its conduct, or interpretation **Low / High / Unclear**
differ from the review question?
*(Low = MRI images were interpreted by a single experienced radiologist and exploratory surgeries were performed by experienced surgeons)
(High = both tests were not performed by appropriate personnel)
(Otherwise code as unclear)*

**Domain 3: Reference standard**

A. Risk of bias

Describe the reference standard and how it was conducted and interpreted:


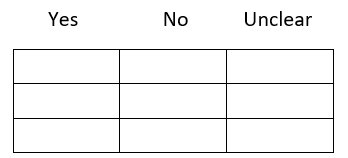


Is the reference standard likely to correctly classify the target condition?
Were the reference standard results interpreted without knowledge of the
results of the index test?

Could the reference standard, its conduct, or its **Low / High / Unclear**
interpretation have introduced bias?
*(Low = answered yes to both)
(High = answered no to both)
(Otherwise code as unclear)*

B. Concerns regarding applicability

Is there concern that the target condition as defined by the **Low / High / Unclear**
reference standard does not match the review question?
*(Low = criteria for root avulsion at surgery was clearly defined and results are reported)*
*(High = no description of the reference standard in the methods and the results alone are reported)
(Unclear = criteria for diagnosis of root avulsion at surgery was unclear or incompletely described in the methods and results are reported)*

**Domain 4: Flow and timing**

A. Risk of bias

Describe any patients who did not receive a preoperative MRI and/or exploration or who were excluded from the 2x2 table:

Describe the time interval and any interventions between index test(s) and reference standard:


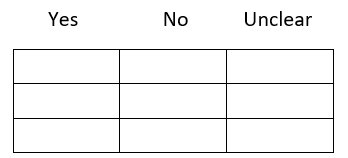


Was the interval between MRI and exploration <12 months?
Did all cases receive the same brachial plexus exploration?
Were all patients included in the analysis?

Could the patient flow have introduced bias **Low / High / Unclear**
*(Low = answered yes to all questions)
(High = answered no to at least one question)
(Otherwise code as unclear)*
